# Theoretical Investigations on Mechanisms and Pathways of C_2_H_5_O_2_ with BrO Reaction in the Atmosphere

**DOI:** 10.3390/molecules23061268

**Published:** 2018-05-25

**Authors:** Chenggang Lu, Yizhen Tang, Wei Zhang, Xunshuai Qu, Zhihao Fu

**Affiliations:** 1 School of Environmental and Municipal Engineering, Qingdao University of Technology, Qingdao 266033, Shandong, China; luchg@qut.edu.cn (C.L.); zhangw@qut.edu.cn (W.Z.); fzh@qut.edu.cn (Z.F.); 2 Qingdao Environmental Monitoring Center, Qingdao 266003, Shandong, China; quxs@qut.edu.cn

**Keywords:** C_2_H_5_O_2_, BrO, atmospheric reaction, mechanism, photolyze

## Abstract

In this work, feasible mechanisms and pathways of the C_2_H_5_O_2_ + BrO reaction in the atmosphere were investigated using quantum chemistry methods, i.e., QCISD(T)/6-311++G(2df,2p)//B3LYP/6-311++G(2df,2p) levels of theory. Our result indicates that the title reaction occurs on both the singlet and triplet potential energy surfaces (PESs). Kinetically, singlet C_2_H_5_O_3_Br and C_2_H_5_O_2_BrO were dominant products under the atmospheric conditions below 300 K. CH_3_CHO_2_ + HOBr, CH_3_CHO + HOBrO, and CH_3_CHO + HBrO_2_ are feasible to a certain extent thermodynamically. Because of high energy barriers, all products formed on the triplet PES are negligible. Moreover, time-dependent density functional theory (TDDFT) calculation implies that C_2_H_5_O_3_Br and C_2_H_5_O_2_BrO will photolyze under the sunlight.

## 1. Introduction

With more and more attention paid to atmospheric environments, researchers focused on the reactions that will increase or produce pollution. Observation indicates halogen monoxides, i.e., *X*O (*X* = Cl, Br, and I) play important roles in the marine boundary layer (MBL) and lower stratosphere [1,2]. As catalysts in the ozone destruction processes, with higher efficiency than ClO, the reactions of BrO with peroxy radicals *R*O_2_ (*R* is organic group), such as HO_2_ and CH_3_O_2,_ have been investigated extensively by experimental and theoretical methods [3,4,5,6,7,8,9,10,11,12,13,14,15,16]. Experimentally, the rate constants of BrO reacting with HO_2_ [8,9,10], CH_3_O_2_ [11,12,15] and C_2_H_5_O_2_ [16] radicals were determined by several groups. For the reactions of HO_2_ and CH_3_O_2_ with BrO, mechanisms and reaction pathways were investigated theoretically [11,12,13,14,16]; however, the products were not confirmed for the C_2_H_5_O_2_ + BrO reaction from experiments, and no literature is available from theoretical investigations yet. The following channels were proposed by Sakamoto [16]

C_2_H_5_O_2_ + BrO → C_2_H_5_O + BrOO   Δ*_r_H* = 0.2 kJ/mol

→ C_2_H_5_O + OBrO   Δ*_r_H* = 56.1 kJ/mol

→ CH_3_CHO + HOOBr   Δ*_r_H* = −222.1 kJ/mol

→ CH_3_CHO + HBr + O_2_   Δ*_r_H* = −294.4 kJ/mol

Due to distinct advantages over experimental methodology, quantum chemistry is popular as a useful tool to explore mechanisms and feasible products in many atmospheric reactions [17,18,19,20]. By quantum chemistry methods, the C_2_H_5_O_2_ + BrO reaction in the atmosphere was explored at the molecular level to address the mechanisms, channels and products. Moreover, it is expected to provide useful information and new insights into the atmospheric chemistry of C_2_H_5_O_2_ with BrO.

## 2. Results

**Figure 1:**
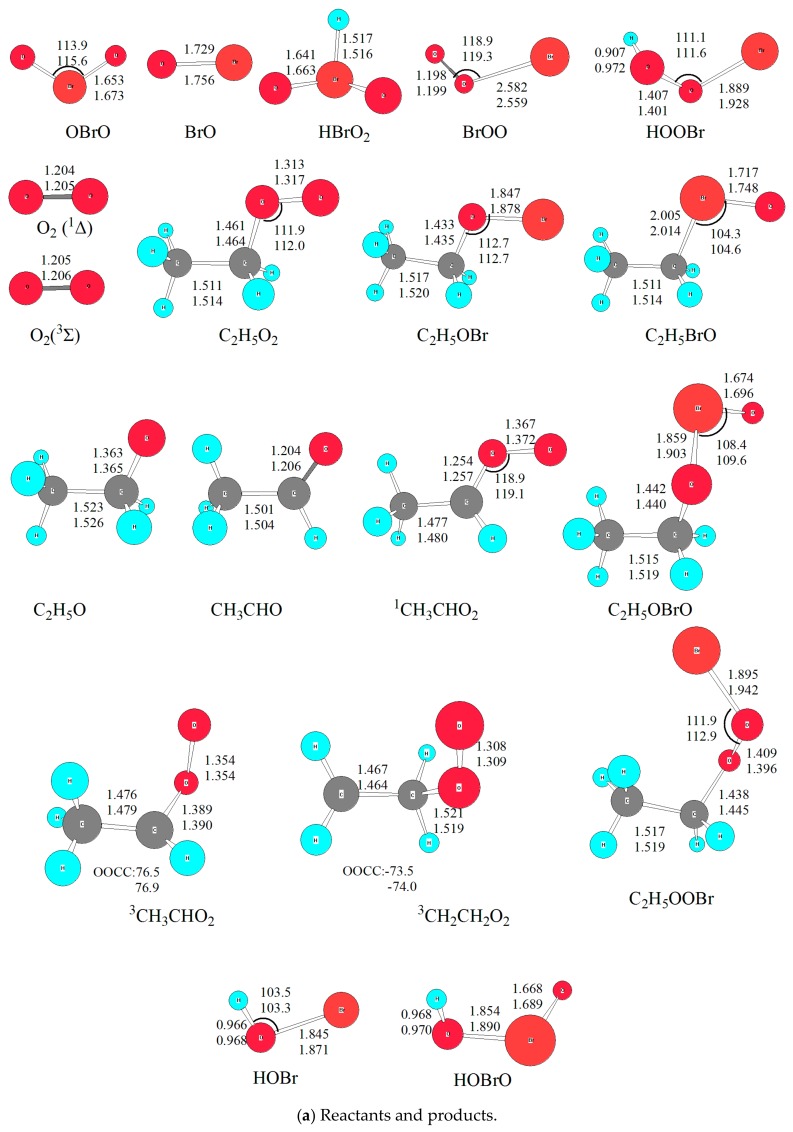

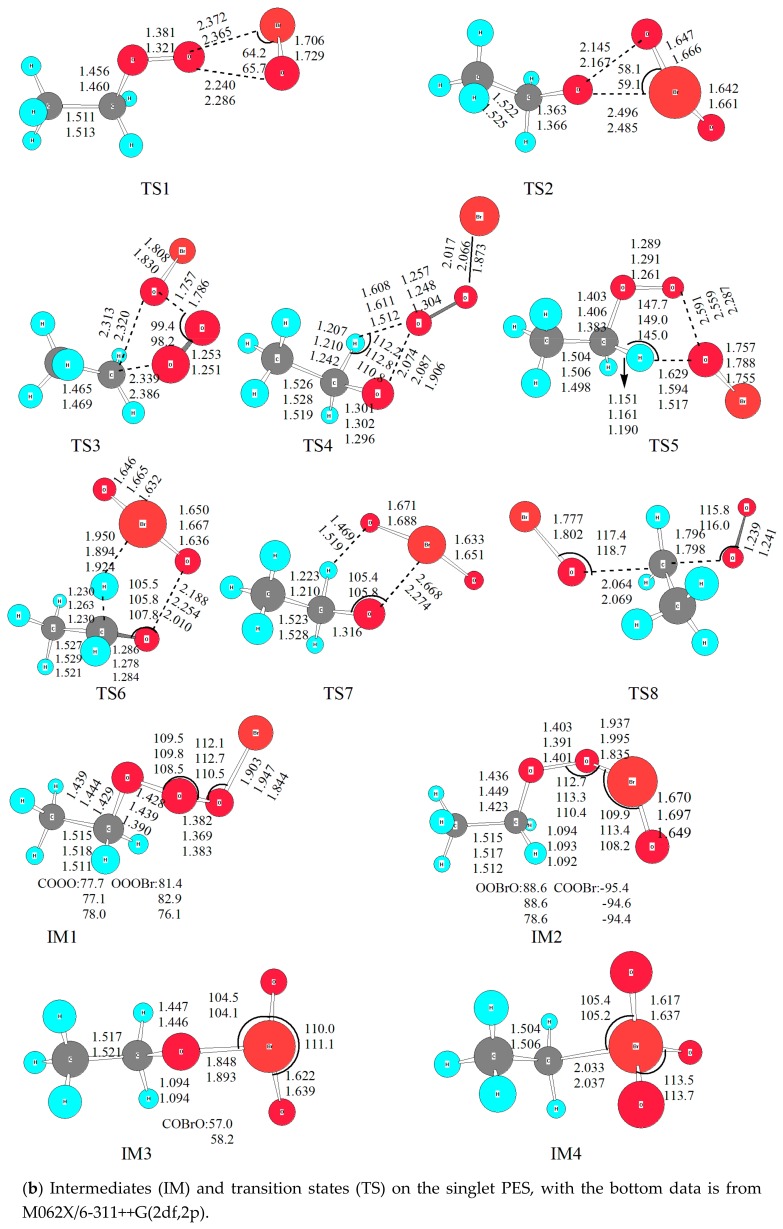

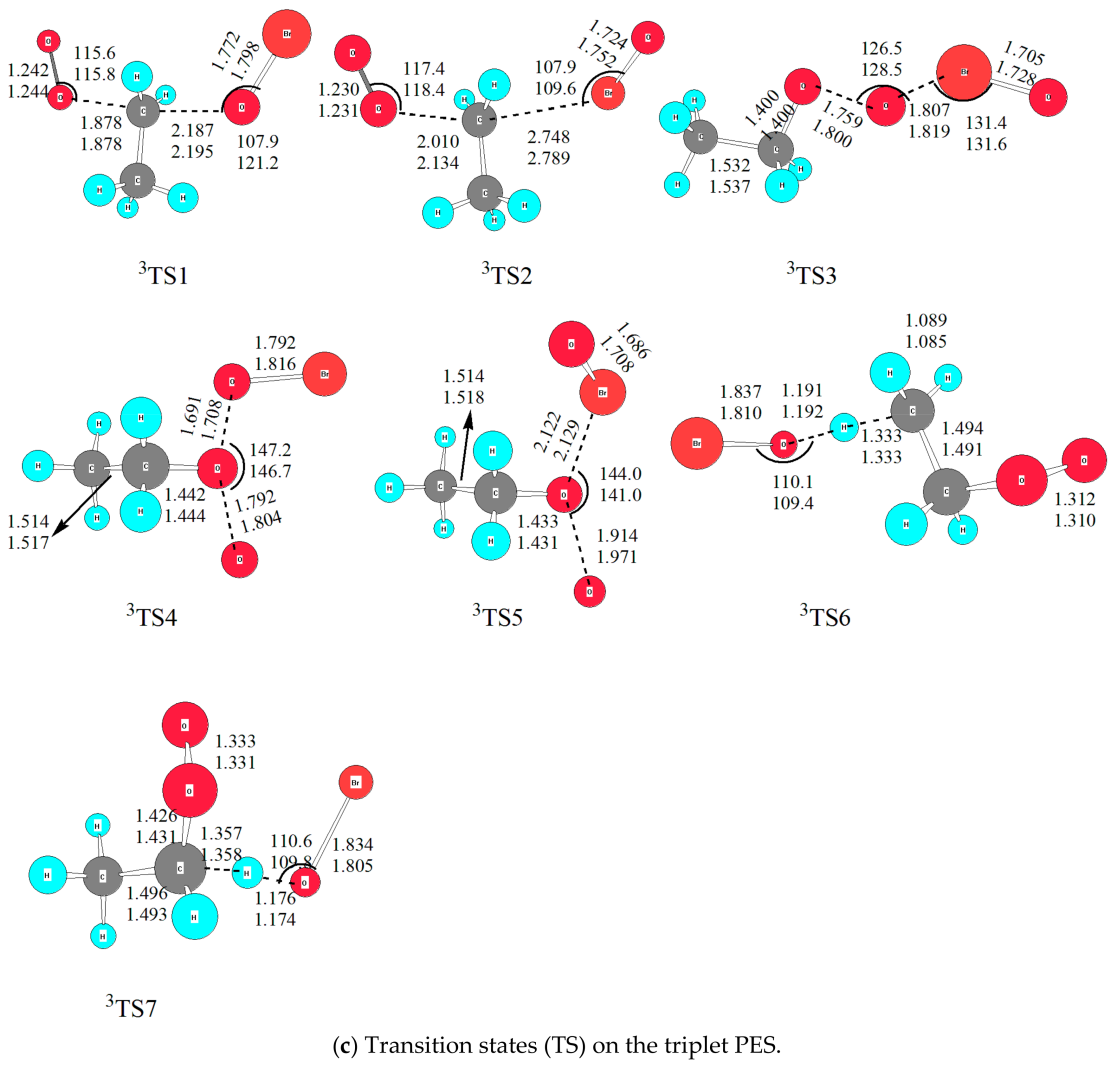
The optimized geometries for all species in the C_2_H_5_O_2_ + BrO reaction at B3LYP/6-311++G(d,p) and B3LYP/6-311++G(2df,2p) levels. Bond distances are in Å and bond angles are in degrees. The upper one is from B3LYP/6-311++G(2df,2p); and the lower one is from B3LYP/6-311++G(d,p). (**a**) reactants and products; (**b**) intermediates (IM) and transition states (TS) on the singlet PES, with the bottom data is from M062X/6-311++G(2df,2p); (**c**) transition states (TS) on the triplet PES.

**Figure 2:**
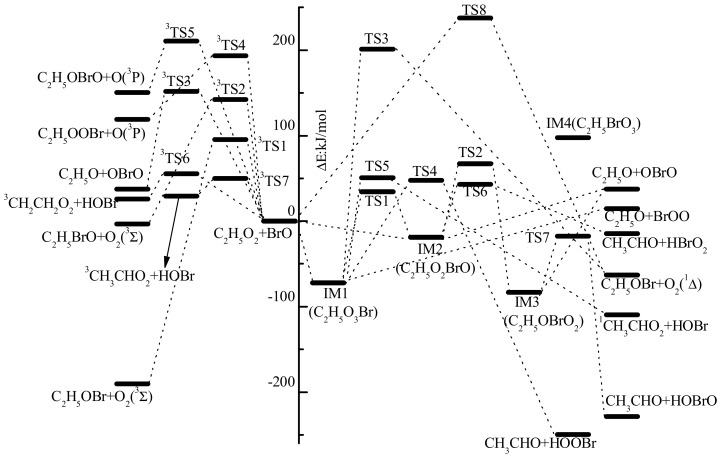
Energetic profiles of the C_2_H_5_O_2_ + BrO reaction at the QCISD(T)/6-311++G(2df,2p)//B3LYP/6-311++G(2df,2p) levels.

Optimized geometries of all reactants, products, intermediates (IM) and transition states (TS) involved in the title reaction were shown in Figure 1. The energetic profiles of the singlet and triplet PESs at the level of QCISD(T)/6-311++G(2df,2p)//B3LYP/6-311++G(2df,2p) was depicted in Figure 2. Moreover, 3 is superscripted to differentiate triplet species from the singlet ones. The reaction enthalpy (ΔH) of various channels in the C_2_H_5_O_2_ + BrO reaction obtained from the present work and available references are listed in Table 1. The zero-point energy correction (ZPE) and relative energy (ΔE) and reaction enthalpy (ΔH) at different levels of theory are displayed in Table 2. Before reaction mechanisms and channels are discussed, it is cautious and meaningful to check the validity of the current theoretical level to ensure that our computational result is conceivable and reliable for the title reaction.

### 2.1. Reliability of Theoretical Methods

The reaction enthalpies (*ÿH*) of four possible channels was proposed by Sakamoto [16]. In order to check the reliability of methods, the reaction enthalpies (Δ*H*) for all proposed channels was calculated at four levels of theory, i.e., G4 [21], QCISD(T)/6-311++G(2df,2p)//B3LYP/6-311++G(d,p), QCISD(T)/6-311++G(2df,2p)//B3LYP/6-311++G(2df,2p), and QCISD(T)/6-311++G(2df,2p)//MP2/6-311++G(2df,2p) [22,23,24,25,26]. The result is listed in Table 1, from which it could be found that relative energy (∆E) and reaction enthalpy (∆H) varies from different levels of theory. Although it is common that the MP2 [27] method provides high quality quantitative prediction for many systems; unfortunately, for the title reaction involving open-shell electrons, it really does not perform well. Since it is known that multireference methods, such as Complete Active Space Self Consistent Field (CASSCF) [28], are more accurate to many atmosphere systems, especially for photo-chemical processes [29,30]. However, due to limited computational resources, we chose QCISD(T)/6-311++G(2df,2p)//B3LYP/6-311++G(2df,2p) for single point energy calculation, as used in previous study for the CH_3_O_2_ + BrO reaction [14]. On the other hand, compared with other levels of theory, the density functional (DFT) method was found to be sufficiently accurate for predicting reliable geometries of the stationary points; at the same time, it is not expensive computationally for scanning PES [14,17,20]. Thus, B3LYP functional was used in this work with two basis sets, i.e., 6-311++G(d,p) and 6-311++G(2df,2p) for all stationary points to check the influence of basis sets on geometry. The optimized geometry was depicted in Figure 1, from which it could be seen that the geometrical parameters from both basis sets are close to each other, indicating that polarization functions have no significant influence on the title reaction. Considering the Br atom involved, the geometry obtained from 6-311++G(2df,2p) basis set was used in the discussion unless otherwise stated. Besides, to check the optimized geometry from B3LYP functional, M062X [31] functional was employed as previously used for the CH_3_O_2_ + BrO reaction. The details are shown in Section 2.2.2. Therefore, it is conceivable to discuss the reaction channels based on the QCISD(T)/6-311++G(2df,2p)//B3LYP/6-311++G(2df,2p) calculations.

### 2.2. Reaction Channels of the C_2_H_5_O_2_ + BrO Reaction

In order to give a clear and distinct description of the reaction mechanisms and pathways, we will discuss the formation of intermediates firstly, and the separate reaction channels on the singlet and triplet PESs subsequently.

#### 2.2.1. The Formation of Intermediates in the C_2_H_5_O_2_ + BrO Reaction

On the singlet PES with two reactive sites of BrO attacking the reactive O atom in C_2_H_5_O_2_, two initial intermediates, namely C_2_H_5_O_3_Br (IM1) and C_2_H_5_O_2_BrO (IM2), are formed directly without any energy barriers. As shown in Figure 1, the newly formed O-O bond is 1.369 Å in IM1, and O-Br bond is 1.995 Å in IM2. It is worth noting that the singlet and triplet PESs intersection commonly takes place in the radical-radical reactions, especially for barrierless entrances [32,33]. However, the transition probability between the singlet and triplet surfaces was not treated explicitly in the present calculations. Our extensive attempts to calculate the single-triple crossing point for the entrance of the C_2_H_5_O_2_ + BrO association have not been successful due to the convergence difficulties of the multireference configuration interactions. The complete active spaces (CAS) calculations with all valence electrons are unaffordable at present. It is noted that the intersystem crossing might be significant in some small organic molecules [34,35], therefore, more rigorous treatment of the singlet-triplet transition has to be reserved for further study.

It is mentioned that several conformers of C_2_H_5_O_3_Br and C_2_H_5_O_2_BrO located according to different dihedral angle of BrOOO (OBrOO) and OOOC (BrOOC) with internal rotations of the relevant O-O and Br-O bonds, and these conformers can interconvert within a few kJ/mol energy barriers; however, the interconversion are out of our focus and not considered in this work.

Energetically, IM1 and IM2 are about 72.3 and 19.1 kJ/mol lower than the initial reactants, respectively. Therefore, C_2_H_5_O_3_Br and C_2_H_5_O_2_BrO should be formed as vibrationally hot molecules:

C_2_H_5_O_2_ + BrO → C_2_H_5_O_3_Br^‡^

→ C_2_H_5_O_2_BrO^‡^

With much internal energy available, the hot molecules of C_2_H_5_O_3_Br^‡^ and C_2_H_5_O_2_BrO^‡^ may experience further isomerization and dissociation before being quenched by collisions. According to our result, three conceivable isomerization scenarios are located on the singlet PES.

Firstly, IM1 and IM2 interconverts via a triangular transition state TS1. The broken O-O bond is elongated to 2.286 Å in TS1, which is about 67% longer than that in IM1; while the formed Br-O bond is stretched by 0.37 Å from its equilibrium distance in IM2. The energy barrier height for IM1 isomerizing to IM2 is about 106.7 kJ/mol, and IM2 convert to IM1 is around 53.5 kJ/mol. With so high energy barriers the interconversion between IM1 and IM2 is unfeasible in the normal atmospheric conditions with a temperature below 300 K.

Secondly, via a similar triangular structure TS2 with bromine atom migrating from the central oxygen atom to the oxygen atom in C_2_H_5_O group, IM2 (C_2_H_5_O_2_BrO) isomerizes to IM3 (C_2_H_5_OBrO_2_). Geometrically, the formed Br-O bond is 2.485 Å in TS2, and the broken O-O bond is dramatically stretched by 0.776 Å from that in IM2. While the two non-reactive Br-O bonds (around 1.66 Å) are close to their equilibrium lengths in IM3 (around 1.64 Å). Energetically, IM3 is rather stable on the singlet PES, with its relative energy (ΔE) of 83.6 kJ/mol lower than the initial reactants; and 64.5 kJ/mol lower than IM2. However, the energy barrier height for IM2 to IM3 reaches 86.4 kJ/mol; apparently, it is difficult to proceed with low temperature (e.g., T < 300 K), although IM3 is the most stable intermediate on the whole singlet PES. Nevertheless, it might happen in high temperature conditions such as combustion, which is out of consideration in the present work.

From previous studies on the CH_3_O_2_ + BrO [14], CF_3_O_2_ + IO [36] and CF_3_O_2_ + ClO [37] reactions, it is assumed that a high energy barrier for IM3 (*R*OBrO_2_) → IM4 (*R*BrO_3_) is surmounted. However, IM4 is rather unstable thermodynamically, therefore, it will not play any significant role in the overall reaction in the atmosphere. For completeness, only IM4 was optimized (C_2_H_5_BrO_3_) without the transition state calculated. As expected, IM4 is unstable, with its relative energy of 97.7 kJ/mol higher than the initial reactants. Based on previous theoretical results and our current calculations [13,14], for all intermediates formed in the RO_2_ + BrO (R = H, CH_3_, C_2_H_5_) reactions, the order of relative stability among the RO_3_Br isomers (i.e., HO_3_Br, CH_3_O_3_Br, and C_2_H_5_O_3_Br) from the most stable to the least stable structure is ROBrO_2_ > ROOOBr > ROOBrO > RBrO_3_. This implies that the substitution of alkyl group has no significant effect in RO_3_Br surfaces.

As for the triplet PES, in spite of many attempts, intermediates were not located at the current levels of theory.

To sum up, four intermediates are formed. Several possible dissociation reaction channels are available with abundant internal energy available from intermediates except for IM4. The details will be described in the following section.

#### 2.2.2. The Reaction Pathways on the Singlet PES

According to our result, seven possible products and eight dissociation channels are determined, i.e., four from IM1, two from IM2 and two from IM3, respectively. To give a clear description, we will discuss the formation of products separately.

(a) C_2_H_5_O + BrOO and C_2_H_5_O + OBrO

The barrierless cleavage of the O-O bond from IM1 leads to C_2_H_5_O + BrOO. While the O-O and O-Br bonds in IM2 and IM3 dissociate to give out C_2_H_5_O + OBrO in the same way. Energetically, the relative energy of C_2_H_5_O + BrOO and C_2_H_5_O + OBrO is 14.9 and 37.5 kJ/mol, respectively. Furthermore, the reaction channels of C_2_H_5_O_2_ + BrO → C_2_H_5_O + BrOO and C_2_H_5_O_2_ + BrO → C_2_H_5_O + OBrO are endothermic by 18 and 38.8 kJ/mol, thus they are unfeasible to occur in the atmospheric conditions with low temperature (e.g., T < 300 K).

(b) CH_3_CHO + HOOBr

With migration of H atom in -CH_2_ to the O atom in -OOBr, and the relevant O-O bond fission from IM1, HOOBr + CH_3_CHO will be generated via TS4. The barrier energy height takes a value of 120 kJ/mol, while TS4 is 47.7 kJ/mol on the singlet PES. Moreover, the channel of C_2_H_5_O_2_ + BrO → CH_3_CHO + HOOBr is highly exothermic by 249 kJ/mol, and the product is rather stable thermodynamically. However, considering the low temperature (T < 300 K) in the atmosphere, especially in higher troposphere and lower stratosphere, CH_3_CHO+HOOBr are unfavorable to form kinetically at the current levels of theory.

(c) ^1^CH_3_CHO_2_ + HOBr

Similarly, with migration of the H atom in -CH_2_ to the O atom in -OBr and the relevant O-O bond fission, ^1^CH_3_CHO_2_ + HOBr is generated via a five-membered-ring structure TS5 with a barrier height of 123 kJ/mol. Here it is mentioned that HOBr was presumed in the CH_3_O_2_ + BrO reaction by Shallcross via a much lower energy barrier (around 62 kJ/mol and the transition state is −3.2 kJ/mol) at the CASPT2-F12/AVDZ//M06-2X/AVDZ levels of theory [15]. In order to check the deviation between our present computational results with Shallcross’s, we performed the optimization of several significant intermediates and transition states with M062X functional from DFT methods. The optimized geometrical parameters were listed in Figure 1, from which it could be seen that the bond length and bond angle are close at the B3LYP and M062X methods. Thus, the single point energy deviations come from the employed methods. Regrettably, multiconfigurational methods were not affordable at the moment due to limited computational resource.

(d) CH_3_CHO + HBrO_2_

Starting from IM2, CH_3_CHO + HBrO_2_ (ΔE = −14.8 kJ/mol) is obtained with migration of one H atom from -CH_2_ to Br atom and cleavage of the O-O bond simultaneously via TS6 while the relative energy is about 5 kJ/mol lower than that of TS4. Although the relative energy is close (within 8 kJ/mol) among TS4, TS5 and TS6, the energy barrier height of TS6 is 61.9 kJ/mol, which is much lower than that of TS4 (120 kJ/mol) and TS5 (123 kJ/mol). Thus, the channel via C_2_H_5_O_2_ + BrO → IM2 → TS6 → CH_3_CHO + HBrO_2_ will be more feasible to occur kinetically than the channels via C_2_H_5_O_2_ + BrO → IM2 → TS4 → CH_3_CHO + HOOBr or C_2_H_5_O_2_ + BrO → IM2 → TS5 → ^1^CH_3_CHO_2_ + HOBr. However, all the channels are unfavorable at low temperature in the typical atmospheric conditions (T < 300 K).

(e) CH_3_CHO + HOBrO

From IM3, H atom in -CH_2_ moves to O atom in OBrO forming a rather stable product CH_3_CHO + HOBrO, and this process is exothermic by 227.8 kJ/mol. Although TS7 (ΔE = −17.6 kJ/mol) is the lowest transition state on the singlet PES, the channel via C_2_H_5_O_2_ + BrO → IM2 → TS2 → IM3 → TS7 → CH_3_CHO + HOBrO will make a minor contribution to the overall reaction due to the high energy barrier of TS2 (ΔE = 67.3 kJ/mol).

(f) C_2_H_5_OBr + ^1^O_2_

From IM1, a concerted process occurs via TS3 with the original C-O and O-O bonds stretched to be 2.386 and 1.786 Å, respectively; and the new C-O bond formed with the equilibrium distance to be 2.320 Å. The cleavage of stable C-O and O-O bond is rather tough with the energy barrier height reaching to 273.6 kJ/mol.

Besides the elimination channel from IM1, C_2_H_5_OBr + ^1^O_2_ could be formed via the substitution mechanism as well on the singlet PES, with O atom in BrO attacking the C center in -CH_2_ group via a rather high energy barrier of TS8, which relative energy is around 237.6 kJ/mol. Evidently, both channels have no possibility of occurring kinetically in the normal atmospheric conditions, and are negligible to the overall reaction although the formation of the product is exothermic.

In summary, on the singlet PES C_2_H_5_O_3_Br and C_2_H_5_O_2_BrO will be dominant products. Other minor products include CH_3_CHO + HOOBr via IM1 and TS4, CH_3_CHO + HBrO_2_ via IM2 and TS6, and ^1^CH_3_CHO_2_ + HOBr via IM1 and TS5. Considering the typical limitation of 20 kJ/mol for atmospheric reactions, these sub-dominant channels are of no significance kinetically, although their formations should be feasible thermodynamically.

#### 2.2.3. The Substitution and Abstraction Channels on the Triplet PES

On the triplet PES, no intermediate was located with many attempts, thus the channels are much simpler than that on the singlet PES. According to our result, both substitution and direct abstraction mechanisms were determined leading to seven products. As shown in Figure 2, surmounting ^3^TS1, ^3^TS2, ^3^TS3, ^3^TS4 and ^3^TS5, C_2_H_5_OBr + ^3^O_2_, C_2_H_5_BrO + ^3^O_2_, C_2_H_5_O + OBrO, C_2_H_5_OOBr + O(^3^P) and C_2_H_5_OBrO + O(^3^P) are generated, and the relative energy is −190.5, −3.3, 14.9, 119.1 and 150.2 kJ/mol, respectively. The energy barriers of five transition states are 95.7, 142.4, 151.8, 193.2 and 210.6 kJ/mol, respectively. Obviously, with so high barriers it is conceivable that all channels play no important roles to the title reaction under the atmospheric conditions below 300 K.

Here it is mentioned that the formation of HOBr was located via direct hydrogen-abstraction channels with H atom in -CH_3_ or -CH_2_ group was abstracted. The corresponding transition states are ^3^TS6 and ^3^TS7, with similar relative energies, i.e., 55.4 and 49.6 kJ/mol, respectively, which are modest to happen in higher temperature conditions. The products in the two channels are ^3^CH_2_CH_2_O_2_ and ^3^CH_3_CHO_2,_ which are much more unstable than their singlet species. The formations of ^3^CH_3_CHO_2_ + HOBr and ^3^CH_2_CH_2_O_2_ + HOBr are endothermic by 17.1 and 21.3 kJ/mol, therefore, the channels are unfavorable thermodynamically.

To sum up, from the above discussions it is concluded that all substitution and abstraction channels on both the singlet and triplet PES are of on significance to the C_2_H_5_O_2_ + BrO reaction below 300 K. C_2_H_5_O_3_Br and C_2_H_5_O_2_BrO are dominant to the overall reaction. Thermodynamically, the subsequent dissociation from intermediates leading to CH_3_CHO + HBrO_2_, CH_3_CHO + HOOBr, CH_3_CHO + HOBrO and CH_3_CHOO + HOBr are favorable. However, with high energy barriers involved, these products are difficult to be formed kinetically in the atmospheric conditions below 300 K.

#### 2.2.4. Vertical Excitation Energy *T_V_* of C_2_H_5_O_3_Br, C_2_H_5_O_2_BrO and C_2_H_5_OBrO_2_

It is known that the photo-oxidation of compounds containing bromine is significant for Br atmospheric chemistry, therefore their photolysis might influence the stratosphere and troposphere. In order to obtain new insights of photolytic information into the Br-containing compounds, the vertical excitation energy (*T_V_*) of the first five excited states for C_2_H_5_O_3_Br, C_2_H_5_O_2_BrO and C_2_H_5_OBrO_2_ was calculated by the TDDFT method [38] employing B3LYP/6-311++G(2df,2p), and the results including wavelength (*λ*), excitation energy (*T_V_*) and oscillator strength (*f*) are listed in Table 3.

Known most ultraviolet (UV) light is absorbed and only is 7% left when solar radiation reaches to the surface of Earth, therefore, compounds will be considered to photolyze if the *T_V_* value is smaller than 4.13 eV (about 300 nm of threshold in the visible light). From Table 3 it is seen that the *T_V_* value of the first two/three excited states of C_2_H_5_O_3_Br and C_2_H_5_O_2_BrO take values smaller than 4.13 eV, and their oscillator strength is not null, implying that the C_2_H_5_O_3_Br and C_2_H_5_O_2_BrO photolyze under the sunlight. Checking the occupied and virtual molecular orbitals it is found that the most contribution comes from HOMO-1 to LUMO with an np_O_ → π* transition associated in both C_2_H_5_O_3_Br and C_2_H_5_O_2_BrO. Therefore, it is speculated that the processes C_2_H_5_O_3_Br → C_2_H_5_O_2_ + BrO and C_2_H_5_O_2_BrO → C_2_H_5_O_2_ + BrO occur after absorption of sunlight. On the other hand, the *T_V_* value of all excited states for C_2_H_5_OBrO_2_ is larger than 4.13 eV, thus it will not be the source of reactive bromine species in the troposphere.

## 3. Materials and Methods

All calculations were carried out using GAUSSIAN 09 program package [39]. The geometries of reactants (R), products (P), intermediates (IM), and transition states (TS) involved in the title reaction were optimized using B3LYP [23] and M062X [31] functionals from DFT methods, and two basis [24,25,26] sets, namely, 6-311++G(d,p) and 6-311++G(2df,2p). In order to obtain more reliable relative energy of each stationary point on the potential energy surfaces (PES), single-point energy is refined by QCISD(T)/6-311++G(2df,2p) [22] basis set based on the B3LYP/6-311++G(2df,2p) geometry and G4 method [21]. Moreover, the vertical excitation energy was calculated with TD-B3LYP/6-311++G(2df,2p) [38] level of theory.

## 4. Conclusions

Using quantum chemistry methods, the reaction mechanisms and pathways for the atmospheric reaction of C_2_H_5_O_2_ + BrO were studied in detail at the QCISD(T)//B3LYP levels of theory. The result indicates that the title reaction occurs on both the singlet and triplet PES, with addition-elimination, substitution and direct H/O-abstraction mechanisms involved. The energy barriers on the singlet PES are lower than that on the triplet PES. C_2_H_5_O_3_Br and C_2_H_5_O_2_BrO is dominant on the singlet PES. Thermodynamically, CH_3_CHO_2_ + HOBr, CH_3_CHO + HOBrO, and CH_3_CHO + HBrO_2_ are feasible, while they are of no significance due to high energy barriers. Moreover, C_2_H_5_O_3_Br and C_2_H_5_O_2_BrO will photolyze under the sunlight, which might be one source of Br-containing species in the atmosphere.

## Figures and Tables

**Table 1:** The reaction enthalpies (ΔH) (in kJ/mol) of several channels in the C_2_H_5_O_2_ + BrO reaction obtained form at the various levels of theory.

C_2_H_5_O_2_ + BrO Reaction Channels	G4	QCISD(T)		
	ΔH	ΔH ^a^	ΔH ^b^	ΔH ^c^
C_2_H_5_O + BrOO	−6.8	17.2	18.0	−19.4
C_2_H_5_O + OBrO	30.0	39.3	38.8	36.0
C_2_H_5_OBr + O_2_(^3^∑)	−190.9	−186.3	−189.9	−194.1
C_2_H_5_OBr + O_2_(^1^∆)	−72.8	−62.1	−62.8	−67.9
HOBr +CH_3_CHO_2_	−116.7	−109.1	−108.9	−110.5
HOOBr + CH_3_CHO	−256.4	−247.9	−249.1	−253.8
HBr + CH_3_CHO + ^3^O_2_	−310.0	−313.3	−312.7	−317.1

^a^ QCISD(T)/6-311++G(2df,2p)//B3LYP/6-311++G(d,p); ^b^ QCISD(T)/6-311++G(2df,2p)//B3LYP/6-311++G(2df,2p); ^c^ QCISD(T)/6-311++G(2df,2p)//MP2/6-311++G(2df,2p).

**Table 2:** The Zero-point energy correction (ZPE), relative energies with ZPE including (ΔE) and reaction enthalpy (ΔH) (in kJ/mol) in the C_2_H_5_O_2_ + BrO reaction.

Species	QCISDT		B3LYP
	ΔE	ΔH	ZPE
C_2_H_5_O_2_ + BrO	0	0	191.6
C_2_H_5_O + BrOO	14.9	18.0	179.4
C_2_H_5_O + OBrO	37.5	38.8	179.9
C_2_H_5_Obr + O_2_(^3^∑)	−190.5	−189.9	170.6
C_2_H_5_Obr + O_2_(^1^∆)	−63.3	−62.8	192.5
HOBr + ^1^CH_3_CHO_2_	−109.5	−108.9	188.6
HOOBr + CH_3_CHO	−249.8	−249.1	188.9
C_2_H_5_BrO + O_2_(^3^∑)	−3.3	−1.4	187.3
C_2_H_5_OOBr + O(^3^P)	119.1	119.8	191.3
C_2_H_5_ObrO + O(^3^P)	150.2	151.8	188.9
CH_3_CHO + HBrO_2_	−14.8	−15.1	179.3
CH_3_CHO + HOBrO	−228.6	−227.8	186.5
HOBr + ^3^CH_3_CHO_2_	15.2	17.1	181.2
HOBr + ^3^CH_2_CH_2_O_2_	19.3	21.3	181.9
IM1	−72.3		199.5
IM2	−19.1		197.4
IM3	−83.6		199.2
IM4	97.7		197.1
TS1	34.4		196.7
TS2	67.3		189.8
TS3	201.3		186.0
TS4	47.7		184.1
TS5	50.7		183.6
TS6	42.8		189.5
TS7	−17.6		185.7
TS8	237.6		189.5
^3^TS1	95.7		187.5
^3^TS2	142.4		183.7
^3^TS3	151.8		188.2
^3^TS4	193.2		188.8
^3^TS5	210.6		187.2
^3^TS6	55.4		176.8
^3^TS7	49.6		176.7

**Table 3:** The excitation energy *T_V_* (in eV), oscillator strength *f* (in atomic units) and wavelength *λ* (in nm) of the first five excited states of C_2_H_5_O_3_Br, C_2_H_5_O_2_BrO and C_2_H_5_OBrO_2_ at the TD-B3LYP/6-311++(2df,2p) level of theory.

Excited States	C_2_H_5_O_3_Br			C_2_H_5_O_2_BrO			C_2_H_5_OBrO_2_		
	*T_V_*	*f*	*λ*	*T_V_*	*f*	*λ*	*T_V_*	*f*	*λ*
1	3.02	0.0002	410.2	2.23	0.0000	555.1	4.621	0.0019	268.3
2	3.77	0.0020	328.5	3.94	0.0017	314.4	4.93	0.0240	251.5
3	4.32	0.0046	287.0	4.07	0.1354	304.3	5.11	0.0001	242.8
4	4.57	0.1277	271.2	4.15	0.0019	299.0	5.131	0.0023	241.6
5	5.32	0.0142	233.1	5.74	0.0038	216.1	5.891	0.0712	210.4

## References

[1] Watson R.T., Albritton D.L., Anderson S.O., Lee-Bapty S. (1993). Methyl bromide: Its atmospheric science, technology and economics. Montreal Protocol Assessment Supplement.

[2] Montzka S.A., Reimann S., Engel A., Kruger K., O’ Doherty S.J., Sturges W.T. (2011). Ozone-depleting substances (ODSs) and related chemicals. Scientific Assessment of Ozone Depletion: 2010.

[3] Elrod M.J., Meads R.F., Lipson J.B., Seeley J.V., Molina M.J. (1996). Temperature dependence of the rate constant for the HO_2_ + BrO reaction. J. Phys. Chem..

[4] Bedjanian Y., Riffault V., Poulet G. (2001). Kinetic study of the reactions of BrO radicals with HO_2_ and DO_2_. J. Phys. Chem. A.

[5] Bridier I., Veyret B., Lesclaux R. (1993). Flash photolysis kinetic study of reactions of the BrO radical with BrO and HO_2_. Chem. Phys. Lett..

[6] Poulet G., Pirre M., Maguin F., Ramaroson R., LeBras G. (1992). Role of the BrO + HO_2_ reaction in the stratospheric chemistry of bromine. Geophys. Res. Lett..

[7] Bloss W.J., Rowley D.M., Cox R.A., Jones R.L. (2002). Rate Coefficient for the BrO + HO_2_ Reaction at 298 K. Phys. Chem. Chem. Phys..

[8] Salisbury G., Monks P.S., Bauguitte S., Bandy B.J., Penkett S.A. (2002). A seasonal comparison of ozone photochemistry in clean and polluted air masses at Mace Head Ireland. J. Atmos. Chem..

[9] Creasey D.J., Halford-Maw P.A., Heard D.E., Pilling M.J., Whitaker B.J. (1997). Implementation and initial deployment of a field instrument for measurement of OH and HO_2_ in the troposphere by laser-induced fluorescence. J. Chem. Soc. Faraday Trans..

[10] Sommariva R., Osthoff H.D., Brown B.B., Bates T.S., Ravishankara A.R., Trainer M. (2009). Radicals in the marine boundary layer during NEAQS 2004: A model study of day-time and night-time sources and sinks. Atmos. Chem. Phys..

[11] Aranda A., LeBras G., LaVerdet G.L., Poulet G. (1997). The BrO + CH_3_O_2_ reaction: Kinetics and role in the atmospheric ozone budget. Geophys. Res. Lett..

[12] Enami S., Yamanaka T., Nakayama T., Hashimoto S., Kawasaki M., Shallcross D.E., Nakano Y., Ishiwata T. (2007). A gas-phase kinetic study of the reaction between bromine monoxide and methylperoxy radicals at atmospheric temperatures. J. Phys. Chem. A.

[13] Larichev M., Maguin F., LeBras G., Poulet G. (1995). Kinetics and Mechanism of the BrO + HO_2_ Reaction. J. Phys. Chem..

[14] Guha S., Francisco J.S. (2003). An ab initio study of the pathways for the reaction between CH_3_O_2_ and BrO radicals. J. Chem. Phys..

[15] Shallcross D.E., Leather K.E., Back A., Xiao P., Lee E.P.F., Ng M., Mok D.K.W., Dyke J.M., Hossaini R., Chipperfield M.P. (2015). Reaction between CH_3_O_2_ and BrO radicals: A new source of upper troposphere lower stratosphere hydroxyl radicals. J. Chem. Phys..

[16] Sakamoto Y., Yamano D., Nakayama T., Hashimoto S., Kawasaki M., Wallington T.J., Miyano S., Tonokura K., Takahashi K. (2009). Atmospheric chemistry of BrO radicals: Kinetics of the reaction with C_2_H_5_O_2_ radicals at 233–333 K. J. Phys. Chem. A.

[17] Shao Y.X., Hou H., Wang B.S. (2014). Theoretical study of the mechanisms and kinetics of the reactions of hydroperoxy (HO_2_) radicals with hydroxymethylperoxy (HOCH_2_O_2_) and methoxymethylperoxy (CH_3_OCH_2_O_2_) radicals. Phys. Chem. Chem. Phys..

[18] Wu N.N., Ou-Yang S.L., Li L. (2017). Theoretical study of ClOO + NO reaction: Mechanism and kinetics. Molecules.

[19] De Souza G.L.C., Brown A. (2016). The ground and excited states of HBrO_2_ [HOOBr, HOBrO, and HBr(O)O] and HBrO_3_ (HOOOBr and HOOBrO) isomers. Theor. Chem. Acc..

[20] Vereecken L., Francisco J.S. (2012). Theoretical studies of atmospheric reaction mechanisms in the troposphere. Chem. Soc. Rev..

[21] Curtiss L.A., Redfern P.C., Raghavachari K. (2007). Gaussian-4 theory using reduced order perturbation theory. J. Chem. Phys..

[22] Pople J.A., Head-Gordon M., Raghavachari K. (1987). Quadratic configuration interaction. A general technique for determining electron correlation energies. J. Chem. Phys..

[23] Becke A.D. (1993). A new mixing of Hartree-Fock and local density-functional theories. J. Chem. Phys..

[24] Clark T., Chandrasekhar J., Spitznagel G.W., von Rague Schleyer R. (1983). Efficient diffuse function-augmented basis-sets for anion calculations. Ⅲ. The 3-21+G basis set for 1st-row elements, Li-F. J. Comp. Chem..

[25] Frisch M.J., Pople J.A., Binkley J.S. (1984). Self-consistent molecular orbital methods 25. Supplementary functions for gaussian basis sets. J. Chem. Phys..

[26] Binning R.C., Curtiss L.A. (1990). Compact contracted basis-sets for third-row atoms: Ga-Kr. J. Comp. Chem..

[27] Head-Gordon M., Head-Gordon T. (1994). Analytic MP2 frequencies without fifth order storage: Theory and application to bifurcated hydrogen bonds in the water hexamer. Chem. Phys. Lett..

[28] Wong M.W., Frisch M.J., Wiberg K.B. (1991). Solvent effects 1. The mediation of electrostatic effects by solvents. J. Am. Chem. Soc..

[29] Fang W.H. (1999). A CASSCF study on photodissociation of acrolein in the Gas Phase. J. Am. Chem. Soc..

[30] Fantacci S., Migani A., Olivucci M. (2004). CASPT2//CASSCF and TDDFT//CASSCF mapping of the excited state isomerization path of a minimal model of the retinal chromophore. J. Phys. Chem. A.

[31] Zhao Y., Truhlar D.G. (2006). Comparative DFT study of van der Waals complexes: Rare-gas dimers, alkaline-earth dimers, zinc dimer, and zinc-rare-gas dimers. J. Phys. Chem..

[32] Harvey J.N. (2007). Understanding the kinetics of spin-forbidden chemical reactions. Phys. Chem. Chem. Phys..

[33] Harvey J.N. (2014). Spin-forbidden reactions: Computational insight into mechanisms and kinetics. Comp. Mol. Sci..

[34] Schnappinger T., Kölle P., Marazzi M., Monari A., González L., de Vivie-Riedle R. (2017). Ab initio molecular dynamics of thiophene: The interplay of internal conversion and intersystem crossing. Phys. Chem. Chem. Phys..

[35] Marazzi M., Wibowo M., Gattuso H., Dumont E., Roca-Sanjuán D., Monari A. (2016). Hydrogen abstraction by photoexcited benzophenone: Consequences for DNA photosensitization. Phys. Chem. Chem. Phys..

[36] Li H.W., Tang Y.Z., Wang R.S. (2013). Atmospheric chemistry of CF_3_O_2_: A theoretical study on mechanisms and pathways of the CF_3_O_2_ + IO reaction. Phys. Chem. Chem. Phys..

[37] Tang Y.Z., Sun H.F., Sun J.Y., Zhang Y.J., Wang R.S. (2014). Theoretical study on mechanisms and pathways of the CF_3_O_2_ + ClO reaction. Atmos. Environ..

[38] Scalmani G., Frisch M.J., Mennucci B., Tomasi J., Cammi R., Barone V. (2006). Geometries and properties of excited states in the gas phase and in solution: Theory and application of a time-dependent density functional theory polarizable continuum model. J. Chem. Phys..

[39] Frisch M.J., Trucks G.W., Schlegel H.B., Gill P.W.M., Johnson B.G., Robb M.A., Cheeseman J.R., Keith T.A., Petersson G.A., Pople J.A. (2010). Gaussian 09.

